# CXCL12 chemokine dimer signaling modulates acute myelogenous leukemia cell migration through altered receptor internalization

**DOI:** 10.1101/2024.08.26.609725

**Published:** 2024-08-27

**Authors:** Donovan Drouillard, Michael Halyko, Elizabeth Cinquegrani, Donna McAllister, Francis C. Peterson, Adriano Marchese, Michael B. Dwinell

**Affiliations:** 1 Department of Microbiology & Immunology, Medical College of Wisconsin, Milwaukee WI, USA; 2 Center for Immunology, Medical College of Wisconsin, Milwaukee WI, USA; 3 Department of Pediatrics, Medical College of Wisconsin, Milwaukee WI, USA; 4 Department of Biochemistry, Medical College of Wisconsin, Milwaukee WI, USA; 5 Department of Surgery, Medical College of Wisconsin, Milwaukee WI, USA

**Keywords:** Chemokines, CXCR4, Acute Myeloid Leukemia, GPCR, receptor internalization, cell signaling

## Abstract

Acute myeloid leukemia (AML) is a malignancy of immature myeloid blast cells with stem-like and chemoresistant cells being retained in the bone marrow through CXCL12-CXCR4 signaling. Current CXCR4 inhibitors mobilize AML cells into the bloodstream where they become more chemosensitive have failed to improve patient survival, likely reflecting persistent receptor localization on target cells. Here we characterize the signaling properties of CXCL12-locked dimer (CXCL12-LD), a bioengineered variant of the dimeric CXCL12 structure. CXCL12-LD binding resulted in lower levels of G protein, β-arrestin, and intracellular calcium mobilization, consistent with the locked dimer being a partial agonist of CXCR4. Further, CXCL12-LD failed to induce chemotaxis in AML cells. Despite these partial agonist properties, CXCL12-LD increased CXCR4 internalization compared to wildtype and locked-monomer forms of CXCL12. Analysis of a previously published AML transcriptomic data showed CXCR4 positive AML cells co-express genes involved in chemoresistance and maintenance of a blast-like state. The CXCL12-LD partial agonist effectively mobilized stem cells into the bloodstream in mice suggesting a potential role for their use in targeting CXCR4. Together, our results suggest that enhanced internalization by CXCL12-LD partial agonist signaling can avoid pharmacodynamic tolerance and may identify new avenues to better target GPCRs.

## INTRODUCTION

Chemotactic cytokines, known as chemokines, are a highly conserved family of secreted proteins that utilize a conserved multisite binding mechanism to activate their cognate G protein-coupled receptor (GPCRs). Chemokine directed cell movement follows a biphasic response wherein movement occurs in a narrow concentration range and is absent at lower and higher concentrations [1, 2]. While chemotactic migration follows a biphasic dose response, pharmacologic signaling through chemokine activated GPCRs typically induce saturable sigmoidal response curves [3]. Chemokine receptor GPCR signaling includes activation of heterotrimeric G proteins and β-arrestin recruitment to direct cellular migration [4]. The chemokine superfamily consists of over 40 chemokines and 15 chemokine receptors that display considerable promiscuity with multiple ligands acting as agonists at the same receptor. How chemokines initiate selective functions in receptor expressing cells present may be explained by the ability of chemokine GPCRs to signal with different efficacies to diverse downstream signaling pathways. Thus, functional selectivity mediated by ligand agonists may reflect activation of a subset of the GPCRs signaling pathways, termed biased agonism, or the entirety of the receptors G protein and arrestin signaling mechanisms, known as balanced agonism [5, 6].

Wild-type CXCL12 chemokine can self-associate into dimers at high concentrations or following interactions with its cognate receptors or glycosaminoglycan binding partners [7]. To determine the impact of ligand oligomerization on chemokine function the Volkman group has engineered CXCL12 into variants locked into either a dimeric structure, CXCL12-locked dimer (CXCL12-LD) or a monomer form, CXCL12-locked monomer (CXCL12-LM) [8]. These forms are structurally identical to native CXCL12 conformations attained by oligomerization of the wild-type protein and retain the ability to bind to and activate the cognate receptors CXCR4 or ACKR3 [9]. Recognizing that chemotaxis typically follows a biphasic response as chemokine ligand levels increase and that dimerization increases as concentration increases, we initially used locked monomer and dimer variants to uncover a role for the monomer form of CXCL12 to initiate cell migration while the dimer was incapable of producing chemotaxis and induced a stationary phenotype we have termed “ataxis” [1, 10]. Subsequently, we discovered that while both CXCL12 monomers and dimers stimulated calcium mobilization, the monomer more potently recruited beta-arrestin [7, 9]. Further, we demonstrated that functionally selective biphasic migration induced by CXCL12, a chemokine originally defined as stromal-derived factor-1 based on its abundant production by bone marrow stromal cells [11], possessed biased agonist signaling of myosin-light chain and filamentous-actin in pancreas cancer cell lines [10, 12, 13]. Thus, our data from cell culture models suggest that CXCL12 induced chemotaxis, with maximal migration observed in a narrow concentration range of ligand, reflects formation of dimers. *In vivo*, CXCR4-mediated chemotaxis regulates the trafficking of hematologic cells into primary and secondary lymphoid tissues, angiogenesis, and in cancer as a key regulator of metastasis [14, 15]. While CXCR4 and CXCL12 expression has been correlated with CXCR4-driven metastasis in solid cancers, its role in hematological malignancies remains less well characterized. This gap may reflect that CXCR4 is thought to function less in chemotactic migration and more as a retention signal that keeps malignant cells within the bone marrow where they are protected from chemotherapy [16]. While we have previously dissected CXCL12 signaling in colon, breast, and pancreas solid tumors, the pharmacologic properties of monomeric and dimeric ligands in hematological malignancies, such as acute myeloid leukemia (AML), remains unknown.

AML is a heterogenous malignancy of adults and children characterized by clonal expansion of immature myeloid blast cells with resulting bone marrow failure and ineffective erythropoiesis [17]. The standard of care chemotherapy during induction therapy consists of chemotherapies daunorubicin and cytarabine and is relatively effective in younger patients [18], with a 5-year survival rate of 55%, but ineffective in older patients with a 5-year survival rate of only 17% in patients greater than 60-years-old due to a lower tolerance for high-dose chemotherapy [19]. With a median diagnosis age of 68 years [20], there is a pressing need to develop new, non-cytotoxic therapies to treat AML in the elderly. An attractive therapeutic target in the leukemia microenvironment is the CXCL12-CXCR4 chemokine axis, which helps maintain AML cells in their protective bone marrow niche [21]. AML cell lines variably increase CXCR4 expression upon chemotherapy treatment, resulting in increased CXCL12-mediated chemotaxis and conferring a bone marrow stroma-mediated survival advantage [22]. In preclinical models with human AML xenografts, AMD3100, a clinically available inhibitor of CXCR4 known by the trade name Plerixafor, combined with chemotherapy demonstrated chemosensitizing effects supporting a role for the CXCL12-CXCR4 axis as a mediator of stromal-dependent chemoresistance [23, 24]. In addition, a number of small peptide CXCR4 antagonists have been studied in preclinical mouse models and have shown similar chemosensitizing effects through a variety of pathways including mobilization of AML cells into the peripheral blood [25], activation of pro-apoptotic pathways [26, 27], or inhibition of proliferation [25]. Despite this preclinical success, phase I/II clinical trials combining AMD3100 with a variety of chemotherapy regimens in adults with AML have found little to no clinical benefit [27–30]. Indeed, further clinical trials with newer generation of CXCR4 inhibitors were, as with AMD3100, terminated due to their lack of efficacy [31, 32]. The failure of current CXCR4 inhibitors highlights the need to better understand the signaling mechanisms whereby CXCL12 and CXCR4 regulate AML functions. Using rationally designed and engineered CXCL12 variants locked into their dimeric structure we have uncovered that the dimer, and not the monomer, is a partial agonist of CXCR4 that stimulates ligand-induced receptor internalization that is stronger and more sustained compared to monomer or wild-type ligand. This internalization occurs independent of chemotaxis as the dimers were unable to stimulate cell migration, despite retention of G protein signaling and β-arrestin recruitment. The sustained internalization of CXCR4 by dimeric ligand suggests a new pathway to disrupt CXCL12-mediated retention of AML within the protective bone marrow niche.

## RESULTS

### Human AML cell lines are CXCR4+ and migrate to CXCL12-WT but not CXCL12-LD

**Figure 1:**
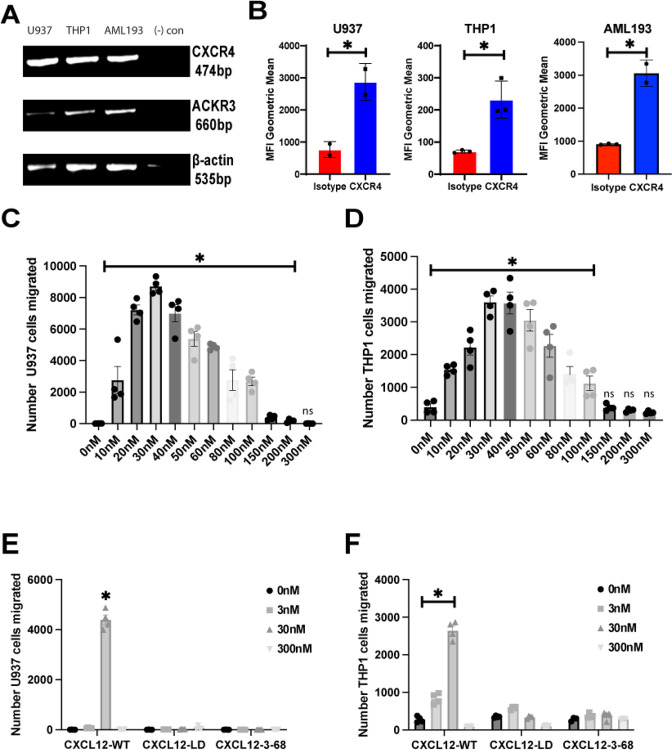
CXCR4 expression in AML cell lines. **(A)** RT-PCR of CXCR4, ACKR3, and beta actin in three human AML cell lines U937, THP-1, and AML-193. **(B)** Flow cytometry results of the three AML cell lines stained with either anti-CXCR4 clone 12G5 or control isotype antibody. * = *P* ≤ 0.05. n = 2–3 independent analyses. Values are mean ± SEM. **(C)** U937 or **(D)** THP-1 cell chemotaxis from the top chamber of a transwell filter towards the lower chamber containing the indicated concentration of CXCL12-WT chemokine. All values under significance bar represent a significant increase compared to 0 nM vehicle treated. **(E)** U937 or **(F)** THP-1 chemotaxis from the top chamber of a transwell chamber towards the lower chamber containing the indicated concentration of CXCL12-WT, CXCL12-LD, or CXCL12-3-68. Migrating cells in panels C-F were enumerated after a two hour incubation using flow cytometry. Values represent mean ± SEM, n = 3 independent biological replicates. * = *P* ≤ 0.05 compared to 0 nM vehicle treated cells.

As a first step to investigate mechanisms of chemokine signaling in AML cells, we used RT-PCR and flow cytometry to assess the expression of CXCR4, the cognate receptor for CXCL12 in three human AML cell lines. Consistent with a prior report, CXCR4 mRNA (Fig. 1A) and protein (Fig. 1B) was highly expressed in three different AML cells [33]. In contrast mRNA levels of ACKR3, an atypical chemokine receptor CXCL12 is known to bind, were lower than CXCR4 in each of the cell lines examined (Supplemental Fig. 1).

Chemokine-induced migration typically follows a biphasic dose response with migration occurring over a narrow concentration range. Accumulating evidence from our lab and others support a model wherein the biphasic migration induced by chemokines like CXCL12 reflects distinct binding modes of monomeric and dimeric forms of the chemokine. In this model chemotaxis reflects receptor activation by ligand monomers, with dimer formation becoming predominant as concentration of the ligand increases and induces nonmigratory signaling we have termed ataxis. Consistent with that model, AML cells undergo dose dependent migration in response to CXCL12-WT protein with an optimal concentration of approximately 30 nM (Fig 1C–D). Higher concentrations resulted in a less robust chemotactic response, which is consistent with the expected bell-shaped chemotactic curve due to possible CXCL12-WT dimerization at higher concentrations. Data from transwell migration assays in two separate AML cell lines indicate that CXCL12-LD was unable to stimulate migration at 3, 30, or 300 nM concentrations (Fig 1E–F). Prior CXCL12 structure-function analysis established a substantial role for the first two amino acids in the ligand amino terminus, Lys1 and Pro2, in receptor activation, with a complete loss of Ca^2+^ flux agonist activity upon deletion or substitution of either residue [34]. Previous work with a CXCL12 variant lacking these first two residues (CXCL12_3–68_) suggest that this molecule binds to CXCR4, albeit with reduced affinity [13, 34]. Consistent with those data, CXCL12_3–68_ failed to induce chemotaxis at any concentration (Fig 1E–F).

### CXCL12 G protein signaling and arrestin recruitment

**Figure 2:**
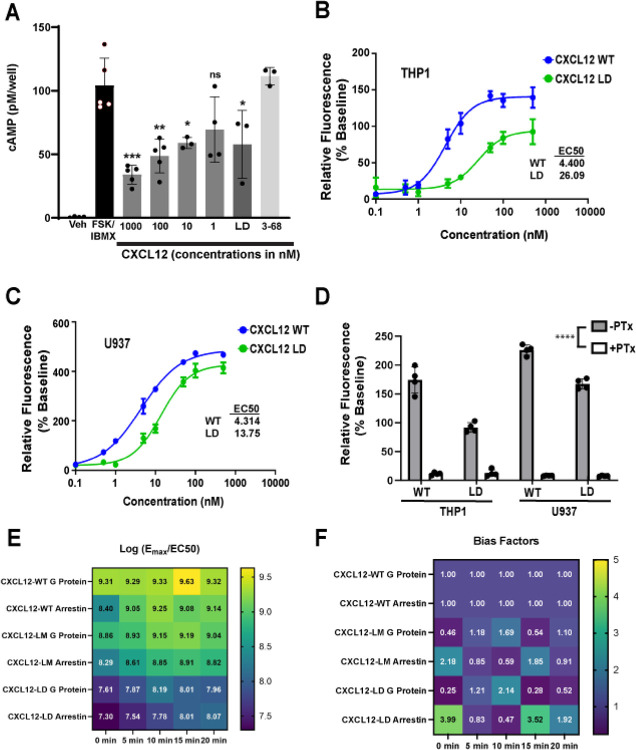
CXCL12-LD is a balanced partial agonist. **(A)** cAMP levels in THP-1 cells stimulated with 10 mM IBMX and 1 μM forskolin followed by treatment with various compounds measured by a competitive enzyme immunoassay. All significance values are in relation to FSK/IBMX. **(B, C)** Calcium flux in THP-1 and U937 cells treated with either CXCL12-WT or CXCL12-LD. n = 3. **(D)** AML cells were pre-treated with pertussis toxin prior to treatment with CXCL12 variants. All pertussis toxin pre-treated values are significantly different when compared to the same form of CXCL12 without pertussis toxin. **(E)** The log(Emax/EC50) of the CXCL12 variants from net BRET in HEK293-CXCR4-Luc cells transfected with β-arrestin or Gαi Venus transducer. n = 6, EC_50_ values calculated from non-linear curve fit, all r^2^ values > 0.82. **(F)** Bias factors for CXCL12-WT, −LM, and −LD at all timepoints tested from HEK293 BRET studies calculated using the calculator found on the Biased Signaling Atlas [35]. A bias factor >5 is indicative of biased agonism.

CXCR4 activates heterotrimeric G proteins, leading to inhibition of adenylyl cyclase and mobilization of intracellular calcium. While CXCL12-induced chemotaxis follows a biphasic dose response, some intracellular signaling pathways such as calcium mobilization and cAMP inhibition exhibit a saturable sigmoidal dose response. As shown in Figure 2, we first assessed G_αi_ signaling in THP-1 cells treated with CXCL12-WT, CXCL12-LD or CXCL12_3–68_. We found CXCL12-WT inhibited cAMP production in a dose-dependent manner with significant inhibition occurring at a concentration of 1000 nM, confirming signaling through the G_αi_ subunit (Fig. 2A). While the dimer was unable to promote chemotaxis, CXCL12-LD inhibited cAMP production at 1000 nM, while CXCL12_3–68_ failed to activate the G protein (Fig.2A).

Next, we measured intracellular calcium flux to study CXCL12 signaling through the other two subunits of the inhibitory G protein: G_β_ and G_γ_. We found that both CXCL12-LD and CXCL12-WT induced intracellular calcium flux in THP1 and U937 AML cell lines (Fig. 2B–C). CXCL12-LD induced calcium flux at lower concentrations than CXCL12-WT. To confirm that the calcium flux was mediated by activation of the GPCR, we performed the same experiment on cells treated in the presence or absence of pertussis toxin, which ADP-ribosylates G_αi_ to prevent the activation of the heterotrimeric G protein. As expected, CXCL12-induced calcium flux was extinguished by the addition of pertussis toxin (Fig. 2D), demonstrating ligand activation of the CXCR4s heterotrimeric G proteins.

In addition to G protein signaling, CXCL12 binding to CXCR4 induces β-arrestin recruitment. Using bioluminescence assays, we measured recruitment β-arrestin to CXCR4 following treatment with either CXCL12-WT, CXCL12-LD, or variant locked into a monomer and unable to form dimers, CXCL12-LM [12]. Compared with wild-type or monomer ligand CXCL12-LD recruited arrestin with less potency over a 20 min time course (Supplemental Fig.2**, left panels**). Similarly, using a mini-G_αi_ BRET we confirmed the lower calcium mobilization evoked by CXCL12-LD and found that the dimer less potently activated the CXCR4 G protein complex (Supplemental Fig.2**, right panels**). Data from the BRET experiments were then used to calculate the transduction efficiencies for each ligand and determined that CXCL12-LD signals as a partial agonist (Fig. 2E). Lastly, the bias factors were calculated for each ligand, with a bias factor of ≥5 indicative of a biased agonist [35], and determined that CXCL12-LD signals a balanced partial agonist while CXCL12-LM is a balanced full agonist in relation to CXCL12-WT (Fig. 2F).

**Figure 3:**
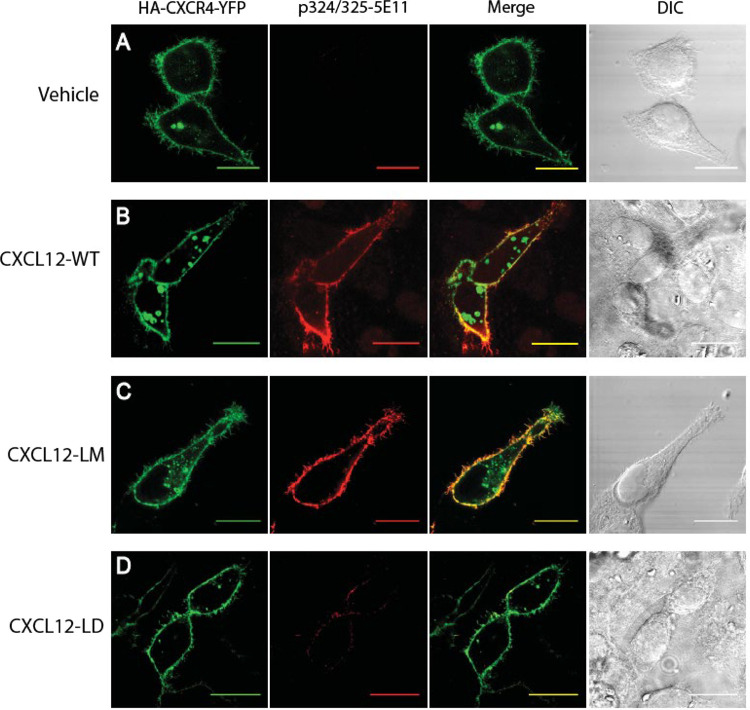
CXCL12-LD shows decreased β-arrestin signaling and GPCR activation through differential phosphorylation. **(A)** HeLa cells transiently transfected with HA-CXCR4-YFP were stimulated with vehicle, **(B)** CXCL12-WT, **(C)** CXCL12-LM, or **(D)** CXCL12-LD. The far left panels show visualization of YFP-CXCR4 and the middle left panel is visualization of the phosphorylated Ser324/325 residues on CXCR4. Co-localization of the YFP-CXCR4 and phosphorylated CXCR4 appear yellow in the merge panel on the middle right. Differential image contrast (DIC) images are shown on the far right panels. Shown are the representative micrographs from three independent experiments. Bars = 20 μm.

β-arrestin recruitment requires phosphorylation of Ser/Thr residues in the CXCR4 C-terminus. The CXCR4 C-terminus has 18 Ser/Thr residues whose site-specific phosphorylation is dynamically regulated by G protein regulatory kinases (GRKs) [36]. As a first step, we used a phospho-specific antibody against dually phosphorylated Ser324 and Ser325 (Ser324/325). Compared to vehicle treated CXCR4 transfectants (Fig. 3A), cells treated with 10 nM CXCL12-WT (Fig. 3B) or CXCL12-LM (Fig. 3C) had increased CXCR4 phosphorylation at Ser324/325. In contrast, CXCL12-LD resulted in weak CXCR4 C-terminal serine phosphorylation (Fig. 3D). Altogether, these data support the notion that CXCL12-LD is a partial agonist that has lower potency and efficacy at signaling through both G_αi_ and β-arrestin.

### CXCL12-LD internalizes CXCR4 more effectively than CXCL12-WT

**Figure 4:**
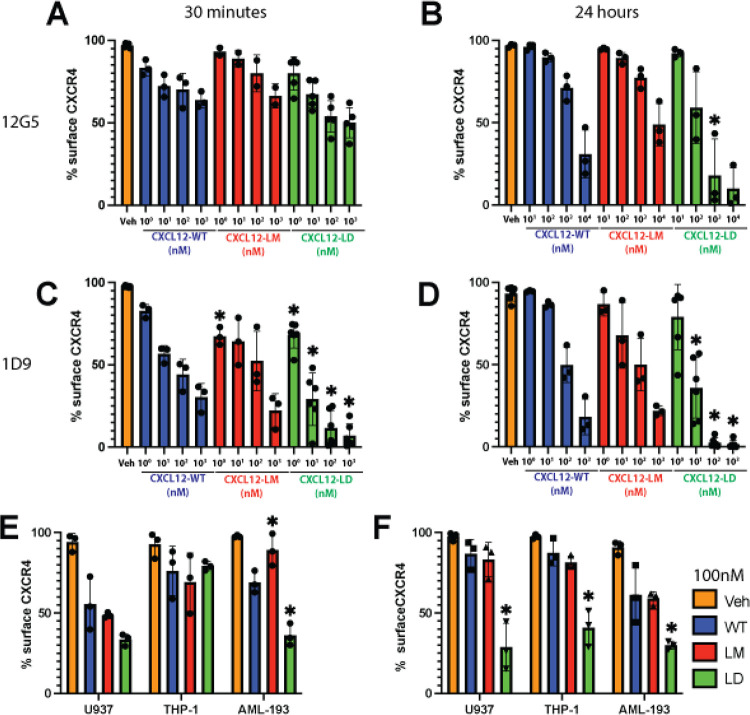
CXCL12-LD promotes internalization of CXCR4. **(A)** Percent of CXCR4 positive U937 cells detected by flow cytometry with the 12G5 CXCR4 antibody after 30 minutes or **(B)** 24 hours of incubation with various concentrations of CXCL12 WT (blue), LM (orange), or LD (green). All values have *P* ≤ 0.05 in relation to untreated cells. Significance represents comparison of CXCL12-variant to respective concentration of CXCL12-WT, *P* ≤ 0.05. **(C)** The percent of CXCR4 positive U937 cells detected by flow cytometry with the 1D9 CXCR4 antibody after 30 minutes or **(D)** 24 hours of incubation with various concentrations of CXCL12 variants. All values have *P* ≤ 0.05 in relation to untreated cells. Significance represent comparison of CXCL12-variant to respective concentration of CXCL12-WT, *P* ≤ 0.05. **(E)** Three different leukimic cell lines treated with CXCL12 WT, LM, or LD and stained with the 1D9 antibody after 30 minutes of treatment or **(F)** 24 hours. Significance represents comparison of CXCL12-variant to CXCL12-WT, *P* ≤ 0.05.

Canonical GPCR internalization occurs largely through β-arrestin dependent signaling mechanisms and has been measured for CXCL12-WT in a variety of cells including human T cells and the rat basophilic leukemic cell line RBL-2H3 [37, 38]. Current antagonists of CXCR4 signaling do not internalize the receptor, likely reflecting their functioning as competitive inhibitors occupying a key ligand binding site [38–40]. Despite the dimer’s limited recruitment of β-arrestin, we measured a significant dose-dependent decrease in CXCR4 surface localization in cells treated with CXCL12-LD at both 30 minutes and 24 hours (Fig. 4A–B). The 12G5 monoclonal antibody binds CXCR4 at the 2^nd^ extracellular loop, a process that may be blocked when different agonists are bound to the receptor [41]. To more stringently evaluate if the receptor was being internalized, we repeated the experiment using monoclonal antibody 1D9, an antibody that binds the N-terminus of the receptor and can therefore detect both ligand-bound and un-bound CXCR4 [42]. Just as with 12G5, we measured decreased surface levels of CXCR4 after 30 minutes (Fig. 4C) and 24 hours (Fig. 4D) of CXCL12-WT, CXCL12-LM, or CXCL12-LD treatment. Both 12G5 and 1D9 antibodies detected a statistically significant decrease in CXCR4 on the surface of cells treated 24 hours with CXCL12-LD compared to CXCL12-WT or CXCL12-LM treated cells. Moreover, 1D9 measured a significant internalization of CXCR4 at 30 minutes that was sustained through 24 hours. CXCR4 internalization was next measured in THP-1 and AML193 cell lines treated with 100 nM CXCL12-WT, CXCL12-LM, or CXCL12-LD and confirmed the larger and more sustained internalization induced by the dimer variant (Fig. 4E–F).

As the three AML cell lines express ACKR3, albeit at lower levels than CXCR4 (Fig. 1) we next asked if the ligand variants modified its internalization. ACKR3 functions as a scavenger receptor that helps sculpt the chemokine gradient without directly invoking cellular migration [43] and is an arrestin-biased receptor in response to CXCL12 [44]. Unlike the internalization measured for CXCR4, none of the CXCL12 oligomers induced internalization of ACKR3 in U937, THP-1, or AML-193 cells (Supplemental Fig. 1).

### CXCL12-LD partial agonist but not CXCR4 antagonists stimulate receptor internalization

**Figure 5:**
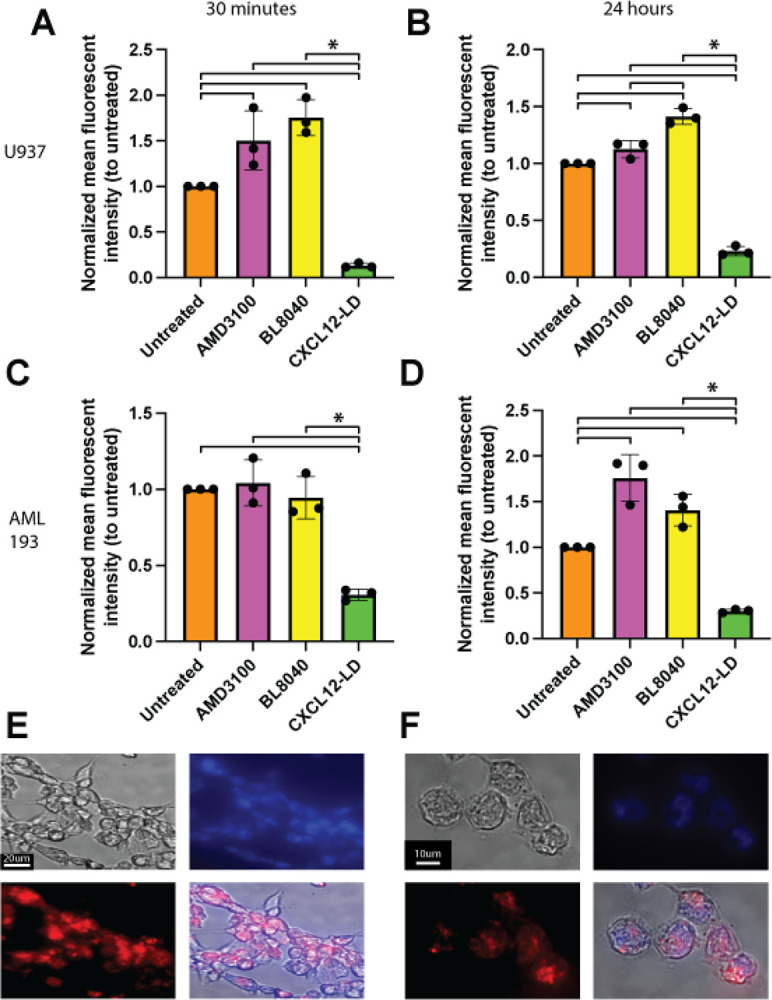
CXCR4 surface upregulation seen with AMD3100 and BL-8040 but not CXCL12-LD. **(A)** U937 cells were treated with either 1 μM AMD3100, 100 nM BL-8040, or 100 nM CXCL12-LD and immunostained for surface CXCR4 with the 1D9 clone. Mean fluorescent intensity (MFI) values were normalized against untreated U937 cells stained for surface CXCR4. Treatment was for 30 minutes or **(B)** 24 hours. **(C)** CXCR4 inhibitor treatment of AML-293 cells for 30 minutes or **(D)** 24 hours under the same conditions seen in 5A and 5B. * = *P* ≤ 0.05. **(E)** HEK293-CXCR4 cells were incubated 24 hours with 25 nM CXCL12-LD conjugated to IR800 (red). Nuceli for individual cells were idenitified using DAPI-staining. Images are representative 40X micrographs or **(F)** 60X micrographs from six different image fields of a single biological replicate.

Previous reports suggest CXCR4 small molecule antagonists induce therapeutic resistance when used to treat patients [28–32]. Conventional wisdom is that therapeutic resistance reflects an upregulation of the receptor by the antagonist [45–49]. To test that notion, we treated U937 (Fig 5A, 5B) or AML-193 (Fig 5C, 5D) cells with AMD3100, BL-8040, or CXCL12-LD and measured CXCR4 surface levels using flow cytometry. An increase in surface CXCR4 was seen with AMD3100 and an even larger increase in surface CXCR4 was seen with BL-8040 in both the 30 minute and 24 hour treatment for the U937 cells (Fig. 5A–B). AML-193 cell showed modest increases in surface CXCR4 after 24 hours of treatment with AMD3100 or BL-8040 (Fig. 5C–D). In stark contrast, CXCL12-LD significantly reduced surface CXCR4 levels in both U937 and AML-193 cells at both timepoints. Lastly, we transfected HEK-293 cells with human CXCR4 (HEK-CXCR4) and separately generated a NIR-fluoro-tagged CXCL12-LD to track internalization to ensure that the decreased surface CXCR4 staining measured by flow cytometry was not due to steric hindrance of the binding site by CXCL12-LD. Cells treated with CXCL12-LD-IR800 for 24 hours demonstrated decreased levels of surface receptor with a corresponding increase in labeled ligand localized within the cytoplasm (Fig. 5E–F). These fluorescence microscopy data support the notion that CXCL12-LD promotes receptor internalization and that our flow cytometry data does reflect the dimer blocking antibody binding sites on CXCR4.

### CXCR4 is upregulated in chemoresistant AML blasts

**Figure 6:**
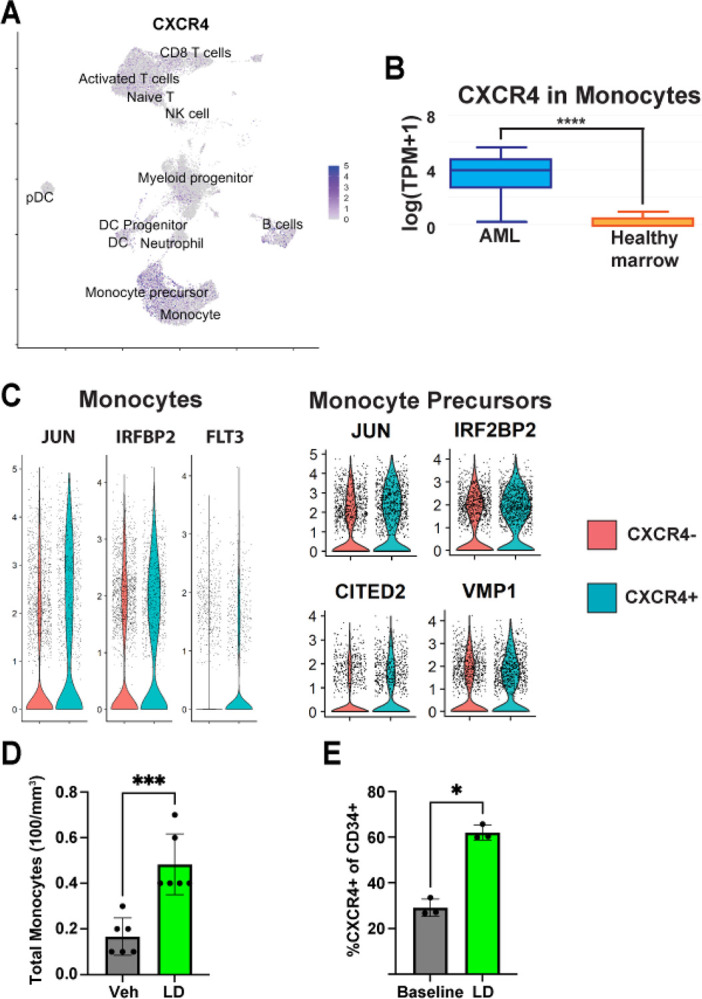
CXCL12-LD increases the frequency of hematopoietic progenitor cells in peripheral blood. **(A)** UMAP of 16 AML patients’ bone marrow aspirates [45]. Purple indicates expression of CXCR4. **(B)** Data analyzed from AML patients in The Cancer Genome Atlas (TCGA) compared to healthy bone marrow data from the Genotype-Tissue Expression Portal (GTEx) using GEPIA2021 [52] comparing CXCR4 expression in AML and healthy monocytes. **(C)** Separate subsets of monocytes and monocyte precursors were sorted based on detectable CXCR4 expression and differentially expressed genes identified using the “FindAllMarkers” function in Seurat. Violin plots of the top results for genes upregulated in the respective CXCR4+ populations (shown in blue). **(D)** Wildtype C57BL/6 mice were given 200 μL 5 μM CXCL12-LD or vehicle PBS. The frequency of total monocytes **(D)** and CD34+ CXCR4+ progenitor cells **(E)** in peripheral blood was measured two hours after treatment. Values are mean ± SD from 3–6 separate mice.

The unique ataxic and internalizing properties of CXCL12-LD prompted further investigation into the potential for the dimer to influence AML *in vivo*. CXCR4 is important for trafficking and retention of hematopoietic cells in the bone marrow [50]. To analyze the CXCR4 expressing cells in AML patients, we turned to publicly available large single-cell RNA datasets. Using a dataset that contains bone marrow aspirates of 16 AML patients and five healthy donors [51], we found CXCR4 expression was enriched in the monocyte precursor and monocyte populations (Fig. 6A). To confirm our findings, we used GEPIA 2021 [52] to analyze expression in monocytes from The Cancer Genome Atlas (TCGA) bulk sequencing data of AML patients and compared it to Genotype-Tissue Expression (GTEx) bulk sequencing data of healthy bone marrow donors. We found a significant upregulation of CXCR4 in monocytes of AML patients compared to healthy donors (Fig. 6B). Next, we wanted to determine the differences between CXCR4 positive and negative cells in AML. We compared the gene expression of CXCR4+ monocytes and monocyte precursors to CXCR4− monocytes and monocyte precursor populations, respectively. We found that CXCR4+ monocytes and monocyte precursors have upregulated expression of the transcription factor JUN and co-factor IRF2BP2 (interferon regulatory factor binding protein two), both of which have been shown to be necessary for AML blast survival [53, 54] (Fig. 6C). Further, CXCR4+ monocyte cells had upregulated expression of FLT3, a frequently mutated gene in AML that is essential for hematopoietic cell survival and is a marker of poor prognosis [55]. The monocyte precursors upregulated the genes CITED2, a transcriptional coactivator important for AML survival, and VMP1, an autophagy protein shown to improve AML cell survival and increase resistance to venetoclax [56–58]. Lastly, to infer the transcriptional regulons driving the CXCR4+ monocyte and monocyte precursor state we completed analyses using the CollecTRI R package [59]. Within the top results in both the CXCR4+ monocyte and monocyte precursor groups were ETV5 and FOXH1, both of which are important for preventing differentiation and maintaining a blast-like state in AML [60, 61] (Table 1). Together these data suggest that CXCR4 is upregulated in AML and that CXCR4+ AML blasts similarly upregulated genes associated with chemoresistance and a stem-like state.

### CXCL12-LD mobilizes monocytes and CXCR4+ hematopoietic stem cells in mice

Small molecular receptor antagonists of CXCR4 are hypothesized to chemosensitize AML cells through mobilization of AML blasts into the peripheral blood [23, 31, 57]. AMD3100 was the first bicyclam CXCR4 antagonist shown to increase the concentration of circulating hematopoietic stem cells in mouse models [24]. To date, however, AMD3100 has had limited success in clinical trials of AML, resulting in no improvement in remission rates compared to historical controls and indicating a need for further exploration of CXCR4 therapeutic targeting [31]. To determine if chemokine oligomers can mobilize CXCR4-expressing cells *in vivo*, wildtype C57BL/6 mice were injected subcutaneously with CXCL12-LD and blood was collected after two hours to assess monocyte mobilization and after four hours to assess CXCR4+ hematopoietic stem cell mobilization. We observed an increase in circulating monocytes in CXCL12-LD treated mice compared to vehicle treated mice (Fig. 6D). Further, there was an increase in the percentage of circulating hematopoietic stem cells that were CXCR4+ (Fig. 6E). These data support the hypothesis that CXCL12-LD, which signals as a partial agonist through CXCR4, promotes long-term mobilization of CXCR4-expressing cells into the peripheral blood and could be used to remove AML blasts out of the chemoprotective bone marrow niche.

## DISCUSSION

Multiple inhibitors of CXCR4 have been developed with varying characteristics. AMD3100 is a 1^st^ generation CXCR4 inhibitor that likely functions as a competitive antagonist. LY2510924 and BL-8040 (Motixafortide, T140, BKT140) are 2^nd^ generation CXCR4 inhibitors and function as inverse agonists with higher potency compared to AMD3100 [62, 63]. CXCL12-LD is an engineered form of the CXCL12 ligand that is structurally and functionally similar to native ligand oligomers and very different compared to current inhibitors of CXCR4. Using reductionist AML cell culture models, we determined that CXCL12-LD, compared to wild-type or monomeric ligand, is a partial agonist of CXCR4. We observed increased internalization of CXCR4 and not ACKR3 with CXCL12-LD with partial agonist G protein and arrestin signaling, with a complete inability to evoke chemotaxis. As both chemotaxis and receptor internalization have been canonically linked with β-arrestin signaling [37] our data suggest a new mechanism for homologous CXCR4 desensitization.

**Figure 7:**
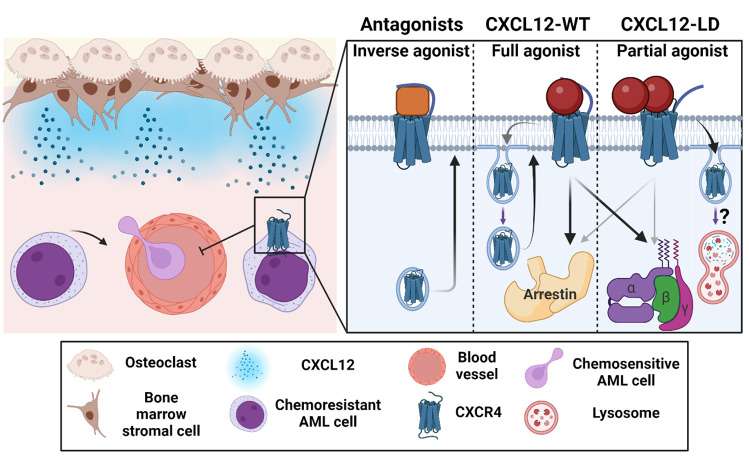
CXCL12-LD functions differently than currently available CXCR4 inhibitors. **Left**, CXCL12 produced by bone marrow stromal cells maintains chemoresistant, CXCR4-expressing AML blasts in the marrow. If CXCR4 expression is lost or the receptor is inhibited, the blasts are mobilized into the bloodstream. **Right**, 2^nd^ generation CXCR4 inhibitors are shown to be inverse agonists of CXCR4 that inhibit internalization and result in surface receptor upregulation. In the middle, CXCL12-WT is a full agonist at both β-arrestin and the G-protein. Signaling with CXCL12-WT results in receptor internalization and recycling. On the right, CXCL12-LD is a non-biased partial agonist. Signaling with CXCL12-LD results in greater receptor internalization compared to CXCL12-WT, possibly through increased receptor lysosomal degradation rather than endosomal recycling. Created with Biorender.com.

Cumulatively, our data summarized in Figure 7 suggest that retention of hematopoietic or transformed cancer cells in the bone marrow may be disrupted by the unique signaling properties of the partial agonist CXCL12-LD. In this study, we show using three different human AML cell lines that CXCL12-LD is a partial agonist of CXCR4. Despite being a partial agonist, our data demonstrate that CXCL12-LD has no chemotactic activity in AML cells. The lack of migration of U937 or AML-193 cells mirrors cellular ataxis we have previously measured in THP-1 cells [12] as well as in colon and pancreas cancer cell lines [1, 10, 12]. Moreover, CXCL12-LD invoked rapid and sustained internalization of CXCR4 but not ACKR3. We show that CXCR4 localization on the surface of AML cells and that CXCR4^hi^ AML cells similarly possess elevated expression of genes involved in treatment resistance, inhibition of differentiation, and cell survival.

Prior studies of CXCL12-WT show it engages with CXCR4 at four different sites on the receptor, or chemokine recognition sites (CRS) [64]. CXCL12-WT binds CRS0.5, 1.0, 1.5, and 2.0 while CXCL12-LD can bind all but CRS0.5 [65, 66]. However, the inability to bind CRS0.5 is thought to only affect signaling efficacy, suggesting that CRS0.5 is required for full ligand agonism [65]. The lack of CXCL12-LD binding to CRS0.5 supports the idea that the decreased CXCR4 1D9 antibody binding measured is not due to steric hindrance by the CXCL12-LD agonist, as the 1D9 antibody clone binds the flexible N-terminus and CRS0.5 is the distal N-terminus of the receptor [67]. Another distinction between CXCL12-LD and CXCL12-WT binding occurs when examining post-translational CXCR4 tyrosine sulfation. Both CXCL12-LD and CXCL12-WT can bind sulfated residues Tyr-21 and Tyr-12 on the CXCR4 amino terminus, while CXCL12-LD differs from wild-type ligand in binding to sulfated Tyr-7 [9, 68]. Additionally, sulfated Tyr-21 binds CXCL12-LD with 20-fold stronger affinity than CXCL12-WT or CXCL12-LM and sulfated Tyr-21 induces ligand dimerization [9]. This gap in knowledge highlights the need to further examine the relationship between receptor tyrosine sulfation, ligand dimerization, and receptor internalization. Interrogation of these structural interactions of chemokine oligomers and their cognate receptors would have the added benefit of improving the design of more effective drugs targeting chemokine receptors, a target that continues to evade successful drug design [69–71].

The receptor internalization measured as concentrations of CXCL12-WT increase after 24 hours suggest the possibility that CXCL12 dimerization may play a key role in mediating CXCR4 internalization. Another alternative explanation for the increased internalization with CXCL12-LD despite decreased β-arrestin recruitment is CXCR4 is internalized in a β-arrestin independent manner. β-arrestin independent internalization of GPCRs has been observed previously and can occur through multiple mechanisms *via* clathrin coated pits [72]. New studies suggest that CXCR4, but not all GPCRs, are internalized through sorting nexins (SNX) and particularly SNX9 and SNX18 [66]. Additionally, the phosphorylated residues within the C-terminus of CXCR4 required to recruit SNX9 are different from those required for β-arrestin. Thus, it is possible that CXCL12-LD differs from monomeric forms of the ligand in its preferential signaling through a pathway requiring SNX recruitment and receptor internalization. Roles for varying CRS elements or tyrosine sulfation in dictating arrestin-dependent or independent mediated internalization has yet to be decoded.

Overexpression of CXCR4 has been demonstrated in a variety of hematologic and solid cancers including AML [22, 73, 74]. While preclinical experiments implicate CXCR4 antagonism as a promising means of chemosensitizing AML and other hematologic malignancies, clinical results have shown limited efficacy [32, 74, 75]. A shared feature of these CXCR4 antagonists is blockade of ligand binding and downstream functional activity. As shown here, AMD3100 and other small molecule antagonists known to inhibit receptor signaling may paradoxically increase receptor surface levels that may in turn overturn the functional blockade sought, a process known as “antagonist tolerance” that can also be seen in other GPCRs. Antagonist tolerance may be a key contributor to treatment resistance and the lack of clinical benefit seen with AMD3100 [45–49, 76–78]. Extrapolating from those *in vitro* data would suggest that AML tolerance to CXCR4 inhibition through persistent surface localization of receptor, occurs within a crucial time-window preceding the initial week-long induction chemotherapy. Using an engineered protein to dissect pharmacological signaling of CXCR4 we have extended our initial discovery of partial agonism in pancreas cancer cells to a high-risk hematologic malignancy to examine how agonists of the same receptor function. The differential ability of CXCL12-LD to promote receptor internalization provides a potential avenue to address roles in pharmacotherapy that traditional antagonists have been unable to address.

## MATERIALS and METHODS

### Cell Lines

Human U937 (CRL-1593.2) and THP-1 (TIB-202) cells were obtained from the ATCC (Manassas, VA) and maintained in RPMI with L-glutamine and 25 mM HEPES (Life Technologies Inc, Carlsbad, CA) supplemented with 10% (v/v) fetal bovine serum (Omega Scientific, Tarzana, CA). HEK-293 epithelial cells (CRL-1573) and HeLa cells (CCL-2) purchased from the ATCC were maintained in DMEM (Life Technologies Inc, Carlsbad, CA) supplemented with 10% (v/v) fetal bovine serum (Omega Scientific). Human AML-193 (CRL-9589) cells were obtained from ATCC and maintained in IMDM supplemented with 5% (v/v) fetal bovine serum, 1X Insulin-Transferrin-Selenium, and 5 ng/mL GM-CSF. Cell lines were authenticated annually using short tandem repeat profiling and mycoplasma-tested semi-annually. HEK293 cells were transfected with HA-CXCR4 using TransIT-LT1 transfection reagents (Mirus Bio, Madison, WI) as previously described [79].

### Recombinant Chemokines

CXCL12 wild-type (CXCL12-WT), locked monomer (CXCL12-LM), locked dimer (CXCL12-LD) and NH2-terminal truncation (CXCL12-3-68) variants were expressed and purified as previously described [12, 80]. The CXCL12-LD mutations are L36C and A65C and create a dimeric protein [8, 13]. The CXCL12-LM mutations are L55C and I58C [12, 76]. The engineered locked dimer or locked monomer proteins are structurally indistinguishable from native dimeric and monomeric protein, respectively, and retain full CXCR4 receptor binding capability [10, 12]. Proteins were expressed in *E. coli* and bacterial cells lysed by French press. Fusion protein was purified through nickel chromatography, refolded by infinite dilution, and ULP1 protease was used to cleave the 6XHIS-Sumo tag. CXCL12-WT and CXCL12-LD were fluorescently labeled with IRDye-800CW (LI-COR, Lincoln, NE) using a two-step process employing sortagging [81] and copper-catalyzed azide-alkyne click chemistry as defined previously [82]. The identity of native or fluorescently labeled CXCL12 was confirmed by linear ion trap quadrupole mass spectrometry. Lyophilized proteins were stored at −20°C.

### Flow Cytometry

THP-1, U937, and AML-193 cells were passaged to 5 × 10^5^ cells/mL the day prior to experiment. Cells were washed twice using cold PBS, incubated in Fc blocking solution (Miltenyi Biotec, Bergisch Gladbach, Germany), and washed twice. Cells were then incubated for 30 minutes in fluorophore conjugated primary CXCR4 antibody (Invitrogen, Waltham, MA) on ice and washed twice. Fluorescence intensity was measured on a BD-LSR II flow cytometer and analyzed by FlowJo software (BD Biosciences, San Jose, CA).

### RT-PCR

RNA was isolated using the RNA Easy kit from Qiagen and treated with DNAse I to remove genomic DNA contaminants. Conversion to cDNA was performed by priming with random hexamers and the SuperScript II cDNA synthesis kit (Life Technologies). PCR products were separated by agarose gel electrophoresis and visualized by ethidium bromide staining and ultraviolet imaging. Primers for actin were used as a loading control. Amplification of chemokine receptors was done using the following primers (5′−3′):

Human CXCR4 Forward 5’-GACTCCATGAAGGAACCCTGTTTCCG-3’

Human CXCR4 Reverse 5’-CTCACTGACGTTGGCAAAGATGAAGTCG-3’

Human β-actin Forward 5’-ACCCACACTGTGCCCATCTACG-3’

Human β-actin Reverse 5’-AGTACTTGCGCTCAGGAGGAGC-3’.

Human ACKR3 Forward 5’-GTGGTGGTCTGGGTGAATATC-3’

Human ACKR3 Reverse 5’-GATGTAGTGCAGGATGGAGAAG-3’

### cAMP Quantification

THP-1 cells were grown in a T-75 flask, then counted immediately prior to the measurement of cAMP using a competitive enzyme immunoassay as we have shown before [83]. THP-1 (5 × 10^5^) cells were aliquoted into 1.5 mL tubes and stimulated with 10 mM 3-isobutyl-1-methylxanthine (IBMX) for 30 minutes at room temperature, after which they were incubated with either CXCL12-WT, CXCL12-LD, or CXCL12-3-68 for an additional 10 minutes. Cells were then stimulated for 10 minutes with 10 μM forskolin (FSK) to stimulate cAMP production. Controls included unstimulated cells and cells treated with IBMX and FSK alone. Cells were then centrifuged and incubated with 0.1 M HCl for 20 minutes before being centrifuged (300 × g on microcentrifuge) for 10 minutes. Supernatant was isolated, and cAMP was assayed using a competitive enzyme immunoassay kit (Cayman Chemical Co., Ann Arbor, MI) according to the manufacturer’s instruction manual. Fluorescence was measured at room temperature with a SpectraMax Multimode Microplate reader (Molecular Devices, San Jose, CA) at a wavelength of 450 nm. Readings were graphed and analyzed according to the immunoassay instruction manual.

### Calcium Mobilization

Calcium mobilization was measured using the FLIPR Calcium 6 assay kit (Molecular Devices) according to manufacturer’s directions. Cells were plated to 50,000 cells/well in 96-well plates. Cells were washed with Calcium/Magnesium-free HBSS supplemented with 20 mM HEPES (Thermo Fisher Scientific, Waltham, MA) and 0.1% (v/v) BSA Fraction V (Thermo Fisher Scientific) and loaded with Calcium 6 dye for 1 hour at 37°C and 5% CO_2_. Chemokines were diluted in calcium/magnesium-free HBSS buffered with 20 mM HEPES and were then loaded onto a separate compound plate. Fluorescence was measured at 37°C with a FlexStation3 Multimode Microplate Reader (Molecular Devices) with excitation and emission wavelengths of 485 and 515 nm, respectively. Chemokines were resuspended at the concentrations indicated in the figure legends and added to the cells after baseline fluorescence was measured for 20 seconds. The percentage Ca^2+^ flux was calculated from the maximum fluorescence minus the minimum fluorescence as a percent of baseline fluorescence. EC_50_ values were determined by nonlinear fitting to a four-parameter logistic function. For experiments with addition of the Gα subunit inhibitor, cells were treated with 100 ng/mL pertussis toxin for 4 hours prior to assessing calcium flux in response to 500 nM CXCL12-WT or CXCL12-LD.

### Confocal Immunofluorescence Microscopy

Phosphorylated CXCR4 serine residues 324 and 325 were detected in HeLa cells as described previously [84], grown on 10 cm dishes were transfected with 5 μg of HA-CXCR4-YFP and passed 24 hours later onto coverslips pretreated with poly-l-lysine, as described previously [85]. Cells were washed once with DMEM plus 20 mm HEPES and serum-starved for 3–4 hours in the same medium, followed by 30-minute treatment with vehicle PBS, 80ng/mL CXCL12-WT, CXCL12-LM, or CXCL12-LD at 37°C. Following treatment, cells were fixed, permeabilized, and immunostained using a custom mouse monoclonal antibody specific for dually phosphorylated serine residues 324 and 325 (pSer324/325) (Clone 5E11), as described previously [84, 86]. Images were acquired using a Zeiss LSM 510 laser-scanning confocal microscope with a 63x W Apochromat oil-immersion objective. Image acquisition settings across parallel samples were identical.

### Transwell Migration

Cells were passaged to 5 × 10^5^ cells/mL the day prior to individual experiments. On the day of the experiment, cells were counted, washed twice, and plated to the upper well of a Transwell insert (Corning Incorporated, Corning, NY) at a density of 60,000 cells/well in assay medium (RPMI-1640, 0.2% (w/v) BSA Fraction V (Thermo Fisher Scientific), with chemoattractants added to the bottom well in assay medium. Cell migration was measured after 2 hours. Transwell migration was enumerated by flow cytometry using the High Throughput Sampler attachment for the BD LSR-II flow cytometer. Results were analyzed with FlowJo software (BD Biosciences).

### BRET Assays

HEK293T cells were passaged to 2 × 10^6^ cells/mL the day prior to the experiment. On the day of the transfection, cell media was changed one hour prior to the introduction of the transfection mixture. OptiMEM was warmed to 37°C and 500 mL was aliquoted into a microfuge tube, along with 0.06 μg Receptor-CXCR4-RLuc8. 5 μg Venus-Transducer β-arrestin and MiniG, and 15.9 μL TransIT. The transfection mixture was incubated in the cell culture hood for 20 minutes and then added dropwise to the cell plates. Cells were then placed back into the incubator overnight.

The day of the BRET assay, cells were washed with PBS and trypsinized for 5 minutes. DMEM supplement with 10% (v/v) FBS was then added to neutralize the trypsin before cells were centrifuged for 5 minutes at 300 × g. Cells were then resuspended in PBS + 0.1% (v/v) glucose. A 15 μL aliquot of cells was placed into a microfuge tube and counted using hemocytometer. Cells should be at a final density of 100,000 cells/well of a 96-well plate. Cells were then plated into a 96-well plate and incubated for 1 hour. Next, the ligand plate was prepared, with the ligands of interest being CXCL12-WT, −LM, and -LD. Ligands were diluted in PBS + 0.1% (v/v) glucose to reach the desired final concentration of 30 μM. Serial dilutions of the ligands were then made in the 96-well plate. Coelenterazine H was resuspended to 1 mM using methanol, and a 50 μL working stock of Coelenterazine H into PBS + 0.1% (v/v) glucose buffer was made. 100 μL of this working stock was added to each well in row A of the 96-well plate. 10 μL of the Coelenterazine H was then transferred from row A to the rest of the rows of the 96-well plate. The Omega plate reader (BMG Labtech; Cary, NC) was then used to measure bioluminescence. Net BRET was calculated using Excel.

### Receptor Internalization

U937, THP-1, and AML-193 cells were counted and placed into 5mL polystyrene tubes at 10^6^ cells/mL in one mL. Cells were treated with the denoted concentrations and variant of CXCL12 for the indicated times. After, the cells were washed twice with 0.5% (v/v) BSA in PBS. The cells then underwent a Fc receptor block (Biolegend 422302) for 10 minutes, washed twice with 0.5% (v/v) BSA in PBS buffer, and then cell surface stained with either PE-conjugated CXCR4 antibody clone 12G5 (Invitrogen 12-9999-42) or PE-conjugated clone 1D9 (BD551510) for 30 minutes. ACKR3 surface levels were measured using ACKR3-APC (Biolegend 331114). After staining, the cells were washed twice with flow buffer and resuspended in 300–500 μL flow buffer. Surface CXCR4 and ACKR3 expression was quantified by analyzing the stained cell suspension on a Cytek Aurora spectral flow cytometer. AMD3100 was used at a concentration of 1uM and BL-8040 was used at 100nM. Results were analyzed with FlowJo software.

### CXCL12-LD-IR800 Imaging

HEK293 cells stably expressing CXCR4 were plated at 25,000 cells per well in a 4 well Falcon chamber slide (Corning 354114) in RPMI +10% FBS. The cells were incubated for 24 hours and then treated with 25 nM CXCL12-LD-IR800 for 24 hours. After 24 hours, the media was aspirated and the cells were washed in PBS and fixed with 4% (w/v) paraformaldehyde for 15 minutes. The cells were then washed three times in PBS, the top of the chamberslide was removed, and the coverslip was applied with two drops of Prolong NucBlue Glass Antifade mount (Thermofisher P36983). Slides were cured for 24 hours and imaged on a Nikon Ti confocal microscope with a 40× and 60× objective (Nikon Instruments, Melville, NY). Image acquisition settings across parallel samples were identical.

### Bioinformatic Analysis

Analysis of a previously published dataset containing 16 AML patients and five healthy donors [51] was performed using the Seurat v4 (4.3.0.1) package in R version 4.3.1 [87]. The data was normalized using the scTransform package [88]. The patient data was integrated using the Harmony package [89] with patient and disease status as covariates. A resolution of 0.6 was used to cluster the cells. Transcriptional regulon analysis was performed using the CollecTRI R package [59]. The code for the analysis can be found at https://github.com/Dwinell-Lab-MCW/Biased-agonist-chemokine-signaling-in-acute-myelogenous-leukemia-cells.

### Mice and Bone Marrow Cell Mobilization

All experiments were conducted under approved protocols from the Medical College of Wisconsin (AUA00076) and in accordance with the National Institutes of Health Guide for the Care and Use of Laboratory Animals and the ARRIVE guidelines [90]. Wildtype C57BL/6J were obtained from The Jackson Laboratory (Bar Harbor, ME). Animals were maintained on a strict 12:12 hour light–dark cycle in a temperature- and humidity-controlled facility with water and food provided ad libitum. Mice were injected with 200 μL 5 μM CXCL12-LD or 200 μL PBS subcutaneously in the right flank. Two hours after injection, mice were euthanized, and blood collected by cardiac puncture. Isolated blood was analyzed on the scil-Vet-abc Hematology Analyzer (scil Animal Care Company GmbH, Viernheim, Germany) to calculate the total number of monocytes per mm^3^. In separate groups of mice, blood was collected from submandibular bleed 4 hours after injection with 200 μL 5 μM CXCL12-LD subcutaneously in the right flank. Red blood cells were lysed using manufacturers protocol (Biolegend 420301) and hematopoietic cells stained with Fixable viability dye eFluor 455 (BD Biosciences 65-0868-14), murine Fc block (Bio X Cell BE0307), murine anti-CD45 PerCP (ThermoFisher MA1–10234), murine anti-CXCR4 SuperBright 600 (ThermoFisher 63-9991-80), and/or murine anti-CD34 FITC (ThermoFisher 11-0341-82) and enumerated on a BD Fortessa flow cytometer (BD Biosciences, Franklin Lakes, NJ).

### Ligand Transduction Coefficients and Bias Calculations

Raw values obtained from individual BRET experiments for both the G protein and β-arrestin recruitment were used to calculate a transduction ratio and bias relative to CXCL12-WT at each time point tested as previously described [87]. To calculate transduction coefficients, CXCL12-LM and CXLC12-LD values were normalized to CXCL12-LD. The Emax and EC_50_ values were calculated using the obtained normalized values. The values were then used to calculate the transduction ratio, or log of the relative activity using log RA = log(Emax/EC_50_). The control-normalized factor was calculate using Δlog RA = log RA _CXCL12 isoform_ – log RA _CXCL12-WT_. The log of the bias factor was calculated using ΔΔlog RA _CXCL12 isoform_= log RA_Pathway1_ – log RA_Pathway2_. Lastly, the final bias factor was calculated using 10^ΔΔlog RA^_CXCL12 isoform_ [35].

### Statistical Analyses

Statistical analyses were performed using Prism 8.0 software (GraphPad Software, Irvine, CA). Power analysis was performed using an alpha error probability of 0.05 and a power level of 0.8 to select rigorous sample sizes for individual experiments. Unpaired sample comparisons between two groups were analyzed by Student’s t-test when data was normally distributed with equal variances of the groups, or by Mann-Whitney test when parametric test conditions were violated. Three or more independent groups were compared using one-way ANOVA with Tukey’s HSD test for multiple comparisons.

## Supplementary Material

Supplement 1

## Figures and Tables

**Table 1.** Transcriptional regulons driving gene expression in CXCR4+ and CXCR4− monocytes and monocyte precursors. The monocyte and monocyte precursor populations from the AML patient dataset presented in Fig. 6 were subset and labeled based on detectable CXCR4 expression [51]. The CXCR4+ and CXCR4− labeled populations were then analyzed using the CollecTRI R package to estimate the transcription factor activity based on weighted mean gene expression in monocytic cell populations in AML patients [59]. The top results are shown for each population, with a positive value representing increased inferred activity and a negative value representing decreased inferred activity.

	CXCR4+ Monocyte	CXCR4− Monocyte	CXCR4+ Monocyte Precursor	CXCR4− Monocyte Precursor
**ASCL2**	1.10	−0.49	0.79	−0.67
**POU3F1**	0.97	−0.43	0.73	−0.61
**FOXC1**	0.93	−0.41	0.70	−0.58
**TBXT**	0.89	−0.39	0.68	−0.57
**ETV5**	0.86	−0.38	0.71	−0.60
**FOXH1**	0.77	−0.34	0.66	−0.55
**KLF2**	−0.45	0.20	−0.32	0.27
**VHL**	−0.58	0.26	−0.41	0.34
**MXD1**	−0.62	0.27	−0.46	0.39
**HDAC3**	−0.74	0.32	−0.53	0.44

## Data Availability

This study did not generate new unique reagents. All data are contained within the manuscript.
