# Supplementary material for: CXCL12 chemokine dimer signaling modulates acute myelogenous leukemia cell migration through altered receptor internalization
Source: bioRxiv. 2024 Aug 27:2024.08.26.609725. Preprint. [Version 1] doi: 10.1101/2024.08.26.609725 (PMC11383031; doi:10.1101/2024.08.26.609725)
Supplement: Supplementary file 1 [file media-1.pdf]

SUPPLEMENTAL INFORMATION

CXCL12 chemokine dimer signaling modulates acute myelogenous leukemia cell migration through altered receptor internalization

Donovan Drouillard[1,2,#], Michael Halyko[2,3,#], Elizabeth Cinquegrani[2,#], Donna McAllister[1,2], Brian F. Volkman[4], Adriano Marchese[4], and Michael B. Dwinell[1,2,5,*]

Supplemental Figure 1.

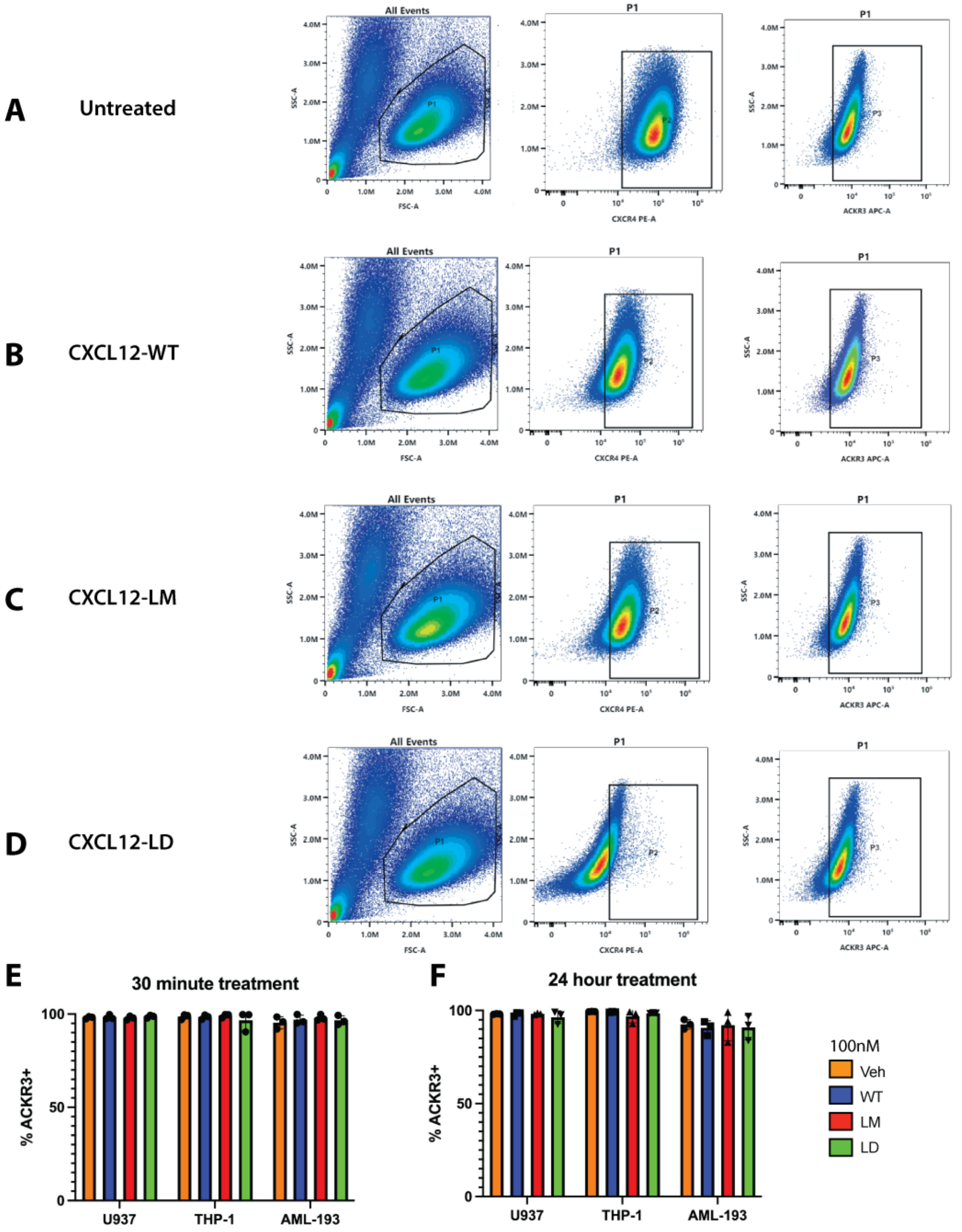

Supplemental Figure 2: Surface ACKR3 levels do not change with any variant of CXCL12. (A) The gating scheme for single cells (left column), CXCR4 surface staining (middle), and ACKR3 surface staining (right) on U937 cells that were untreated, **(B)** treated with CXCL12-WT, **(C)** CXCL12-LM, **(D)** or CXCL12-LD for 24 hours. Flow cytometry representative of 3 independent biological replicates. **(E)** Quantified ACKR3 surface staining on the U937, THP-1, and AML-193 cell lines after treatment with the indicated CXCL12 isoform for 30 minutes or **(F)** 24 hours. No differences in the percent ACKR3 positive cells were detected by one-way ANOVA at either timepoint.

Supplemental Figure 2

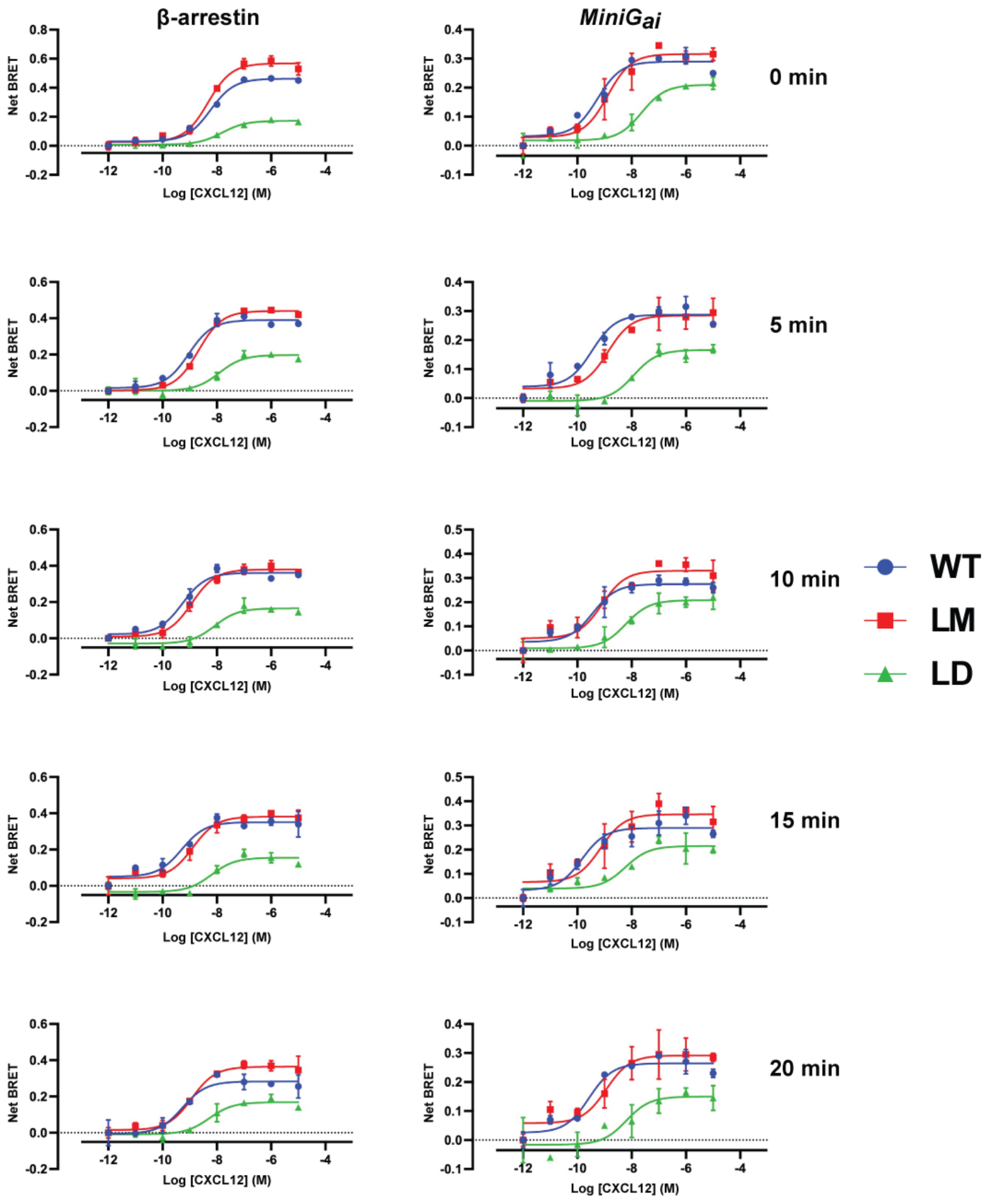

Supplemental Figure 1: G protein and β-arrestin recruitment with CXCL12 variants. Net BRET in HEK293-CXCR4-Luc cells transfected with β-arrestin or Gαi Venus transducer. N = 6, Time points indicate length of incubation with the CXCL12 variant prior to analyzing net BRET. Values obtained from this experiment were used to calculate the values detailed in Figure 2E and 2F.
